# Mesenchymal stem cells cause induction of granulocyte differentiation of rat bone marrow C-kit^+^ hematopoietic stem cells through JAK3/STAT3, ERK, and PI3K signaling pathways

**DOI:** 10.22038/IJBMS.2022.66737.14633

**Published:** 2022-10

**Authors:** Ezzatollah Fathi, Seyed Alireza Mesbah-Namin, Ilja Vietor, Raheleh Farahzadi

**Affiliations:** 1 Department of Clinical Sciences, Faculty of Veterinary Medicine, University of Tabriz, Tabriz, Iran; 2 Department of Clinical Biochemistry, Faculty of Medical Sciences, Tarbiat Modares University, Tehran, Iran; 3 Institute of Cell Biology, Medical University of Innsbruck, Biocenter, Innsbruck, Austria; 4 Hematology and Oncology Research Center, Tabriz University of Medical Sciences, Tabriz, Iran

**Keywords:** BM C-kit^+^ HSCs, Cell therapy, Granulocyte differentiation, Mesenchymal stem cells, Signaling pathways

## Abstract

**Objective(s):**

Hematopoietic stem cells (HSCs) are the cells that give rise to different types of blood cells during the hematopoiesis process. Mesenchymal stromal cells (MSCs) as key elements in the bone marrow (BM) niche interact with hematopoietic progenitor cells (HPCs) by secreting cytokines, which control HPCs maintenance and fate. Here we report that BM-MSCs are capable of inducing granulocytic differentiation of the C-Kit+ HSCs via activating JAK3/STAT3, ERK, and PI3K signaling pathways.

**Materials and Methods:**

For this purpose, BM-MSCs and C-kit^+^ HSCs were isolated. Next, cells were divided into two groups and differentiated into granulocytes: C-kit^+^ HSCs alone (control group) and co-cultured C-kit^+^ HSCs with MSCs (experimental group). Afterward, the gene and protein expression were assessed by real-time PCR and western blotting, respectively.

**Results:**

It was found that BM-MSCs resulted in increased JAK3/STAT3, ERK, and PI3K protein expression in granulocyte differentiated C-kit^+^ HSCs.

**Conclusion:**

It should be concluded that MSCs could affect the granulocyte differentiation of C-kit^+^ HSCs via increasing JAK3/STAT3, ERK, and PI3K signaling pathways.

## Introduction

Mesenchymal stem cells (MSCs) are multipotent cells that can differentiate into different lineage cells including adipocytes, osteocytes, chondrocytes, etc. Today, these cells are isolated from various tissues like adipose, bone marrow (BM), umbilical cord blood (UCB), amniotic fluid, heart, liver, etc. (1, 2). Researchers and clinicians focus on BM as a significant source for experimental purposes. In addition to MSCs, BM is composed of different types of cells such as blood cells, epithelial cells (EPCs), and hematopoietic stem cells (HSCs) (3). The BM microenvironment plays a key role in the proliferation and differentiation of HSCs, MSCs, and stromal cells (4). In the BM, MSCs present a network of cytokines and interact with HSCs which cause physical support for differentiation of these through the secretion of cytokines (5). The role of MSCs in promoting HSCs proliferation and expansion has been reported in previous *in vitro* studies (6, 7). In one study it was pointed out that BM-derived stromal cells cause expansion of BM and CB resident CD34^+^ HSCs. In more detail, the role of different cytokines was proved in the study (6, 8). In 2005, it was first reported that co-transplantation of cultured MSCs and CD34^+^ HSCs could assist engraftment due to the supportive role of MSCs in hematopoiesis (9). In tissue engineering, it was shown that MSCs can repair damaged tissues (10-12). So, these cells are considered effective tools in therapeutic approaches (13). However, the role of different cell types in the BM niche in the differentiation of HSCs is yet to be known. Previous studies indicated that MSCs cause increased myeloid and lymphoid lineage differentiation of progenitor cells (PCs) (14). Chen *et al.* (2013) demonstrated that UCB-MSCs are responsible for the granulocytic differentiation process of primary acute promyelocytic leukemia by secreting interleukin (IL)-6 (15). Following that, Nikkhah *et al.* (2018) pointed out that BM-MSCs increased the CD11b expression in cell line HL-60 and eventually cause granulocyte differentiation (16). 

The role of different signaling pathways in proliferation, differentiation, maturation, and apoptosis of HSCs was previously reported (17). Following the HSCs differentiation, a number of signal pathway proteins such as AKT, Janus family kinases (JAK), signal transducer and activator of transcription (STAT), extracellular signal-regulated kinase (ERK), phosphatidylinositol 3-kinases (PI3K), etc., have been pointed to become activated (18, 19). Investigations related to *in vitro* expansion of HSCs with a cell transplantation approach are among the goals of new research (20). As we know, the BM microenvironment is derived from common progenitors of mesenchymal origin, and MSCs as precursors of cellular components have an effective role in differentiation of HSCs (21). 

This study aims to investigate the role of MSCs in the granulocyte differentiation potential of BM-C-kit^+^ HSCs via some signaling pathways. 

## Materials and Methods

Except for the cell culture materials which were purchased from SPL and Gibco, the rest of the materials are specified in the text of the manuscript.

The cells were divided into two groups: control group (C-kit^+^ HSCs alone) and experimental group (co-cultured C-kit^+^ HSCs with MSCs).

***Isolation of BM-MSCs***

BM-MSCs were isolated as previously reported by Fathi *et al.* (2021).(20) In brief, after obtaining the ethical code, 5 male Rattus norvegicus were euthanized, femur and tibia bones were collected, and BM was flushed. Next, the BM content was layered over the Ficoll-Paque. Mononuclear cells (MNCs) were seeded in 6-well plates containing complete culture media (DMEME low glucose+10% FBS+Pen/Strep). Cells were cultured until they reached 70–80% confluence (22).

***Characterization of BM-MSCs***

BM-MSCs were cultured, trypsinized, and incubated with FITC antibodies CD44, CD90, CD31, and CD34 (BD Phar Mingen, San Diego, CA, USA) in washing buffer (PBS containing 5% FBS). Next, a fluorescence-activated cell sorter was used for characterizing cells (23, 24). 

***Isolation of BM C-kit*** ^+^ ***HSCs***

For isolating the BM-resident C-kit^+^ HSCs, MNCs obtained from the previous step were incubated with C-kit^+^ microbeads (Miltenyi Biotec, Germany). Next, re-suspended cells were passed through one LS MACS column and enriched C-kit^+^ cells were retrieved by flushing the column (25). 

***Characterization of BM C-kit*** ^+^ ***HSCs***

Flow cytometry and immunocytochemistry (ICC) were performed as previously described by Fathi and Farahzadi (2022) for characterizing the BM C-kit^+^ HSCs (24).

***Co-culture of BM C-kit*** ^+^ ***HSCs and BM-MSCs***

To perform the co-culture procedure, BM-MSCs were plated into trans-well plates as two control and experimental groups (20). The granulocyte differentiation of C-kit^+^ HSCs was induced by 50 ng granulocyte colony-stimulating factor (GCSF) (Biolegend, Cat no: 775002). At the end of the 7^th^ day, granulocyte-differentiated C-kit^+^ HSCs were subjected to Real-time PCR analysis for evaluating the granulocyte-related gene expression.

***Gene expression assessment***

At the end of the 7^th^ day, granulocyte-differentiated C-kit^+^ HSCs were collected, total RNA was extracted and cDNA was synthesized. The mRNA expressions of CD11b, CD16, CD18, and CD34 genes were performed using Real-time PCR. Fluorescence data was calculated in relation to *β-actin* CT values by the 2^-ΔΔCT^ method. Primers were listed in Table 1 (26). 

***Western blotting***

At the end of the 7^th^ day, the protein of granulocyte-differentiated C-kit^+^ HSCs was extracted. 50 μg of both groups were loaded to SDS-PAGE and transferred to polyvinylidene difluoride (PVDF) membranes. Next, membranes were incubated with primary antibodies β-actin (1:1000, sc-69879), AKT (1:1000, E-AB-30471), ERK 1/2 (1:1000, sc-292838), p-ERK 1/2 (1:1000, sc-16981-R), JAK3 (1:1000, sc-6932), STAT3 (1:1000, sc-8019), and PI3K (1:1000, sc-8010), and incubated with secondary antibodies (1:5000). Antigen-antibody complex was detected by enhanced chemiluminescence (ECL) reagent (27).

***Statistical analysis***

The results were analyzed using a t-test to determine the significant difference among groups. 

## Results

***Characterization of BM-MSCs***

**Figure 1:**
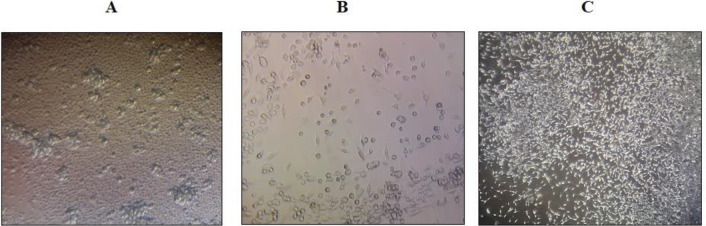
Morphology of bone marrow mesenchymal stem cells. (A) View of cells after isolation from bone marrow (scale bar = 20 μm); (B) Spindle-shaped morphology of cells (bar = 40 μm); and (C) More confluent cells (scale bar = 20 μm)

**Figure 2:**
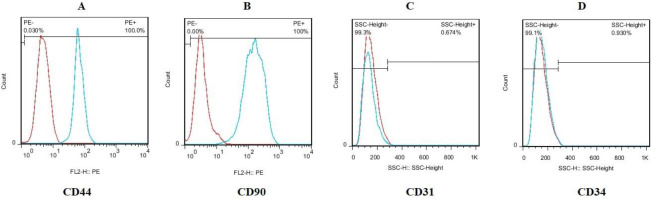
Characterization of bone marrow mesenchymal stem cells. (A) BM-MSCs were positive for (A) CD44 and (B) CD90, and negative for (C) CD31 and (D) CD34. Isotype

BM-MSCs are spindle-shaped fibroblasts (Figures 1A-C). Also, as shown in Figures 2 A and B, the immunophenotypical characterization of BM-MSCs showed high expression levels of CD44 and CD90 and low expression levels of CD31 and CD34 surface markers (Figures 2 C and D).

***Characterization of BM C-kit*** ^+^ ***HSCs***

**Figure 3:**
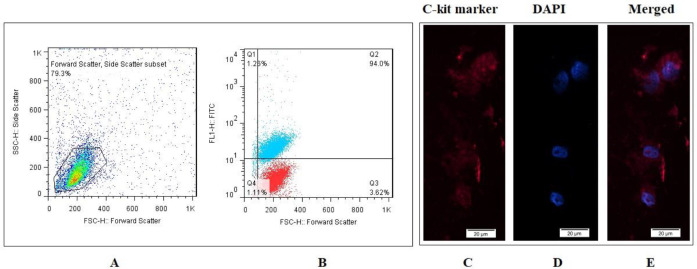
Characterization of isolated bone marrow derived C-kit + cells. (A) Total population of cells for flow cytometry analysis; (B) 94% of cells were positive for C-kit marker; (C-E) Data confirmed the existence of C-kit, indicated by positive color cells by immunofluorescence imaging; Blue = DAPI; Red = PE-conjugated C-kit (bar = 20 μm)

Figures 3A and B show the total cell population and shift of the C-kit^+^ cell population (blue dots) from the isotype control (red blots), respectively. These figures demonstrated that enriched C-kit^+^ cells had high levels of expression of C-kit^+^ (94%). Also, monitoring the protein level of C-kit-related marker by ICC revealed the PE-conjugated C-kit cells (Figures 3C-E). The Fluorescence of the C-kit marker and DAPI staining of nuclei are colored in red and blue, respectively. 

***MSCs cause increased gene expression of granulocyte markers CD11b, CD16, CD18, and CD34***

**Figure 4:**
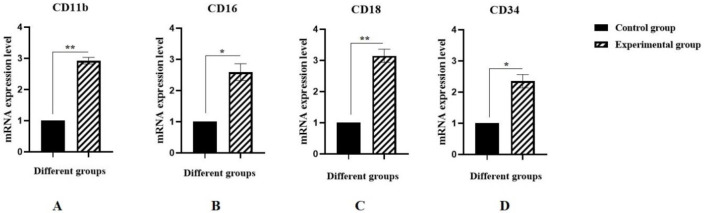
Effect of BM-MSCs on (A) CD11b, (B) CD16, (C) CD18, and (D) CD34 gene expression in granulocyte differentiated BM-derived C-kit^+^ cells. Values are mean ± SD from independent experiments (*P<0.05 and **P<0.01, n = 3)

To investigate the effect of MSCs on the granulocyte differentiation of BM C-kit^+^ HSCs, the gene expression of granulocyte markers was examined (20). The results revealed that gene expression of CD11b, CD16, CD18, and CD34 significantly increased by about 2.92, 2.59, 3.15, and 2.35 times in the experimental group (co-cultured C-kit^+^ HSCs with MSCs) as compared with the control group (C-kit^+^ HSCs alone), respectively (Figure 4). In other words, it could be claimed that MSCs play an important role in the expression of granulocyte markers of the C-kit^+^ HSCs with MSCs. 

***Western blot analysis***

**Figure 5:**
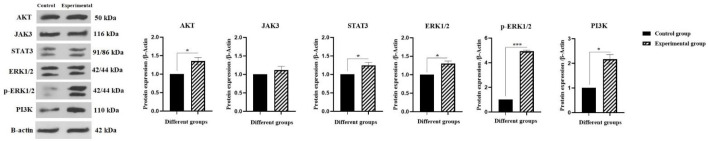
Effect of bone marrow-derived MSCs on expression of AKT, JAK3, STAT3, ERK1/2, p-ERK1/2, and PI3K in granulocyte differentiated BM-derived C-kit+ cells. Values are mean ± SD from independent experiments (*P<0.05 and ***P<0.001)

Following the co-culturing, granulocyte differentiated C-kit^+^ cells were collected and JAK3/STAT3, ERK, and PI3K as signaling components were investigated by western blotting. The expression levels of AKT, JAK3, STAT3, ERK1/2, p-ERK1/2, and PI3K were significantly increased 1.35-, 1.12-, 1.24-, 1.3-, 4.93-, and 2.1-fold, respectively) in the experimental group (Figure 5) (*P<0.05 and ***P<0.001).

**Table 1:** Characteristics of primer sequences used for the Real time-PCR assays

**No.**	**Gene**	**Primer pair sequence (5'-3')**	**Product length (bp)**
NM_012711.1	*CD11b*	AGCCAGTTTCATCAACACAACC GAGGTGCCCCTAAAACCAAGC	116
NM_207603.2	*CD16*	TCTCCAAAAGGCTGTGGTG GACATAGTTGGCGTCCTG	160
NM_001037780.2	*CD18*	ACCTCTCCTACTCTATGC AACGGAGGCTGGCAGGCTT	205
NM_001107202.2	*CD34*	AGACCACACCAGCCATCTC CTTCTGGAGTAGAAGTACTG	153
NM_001101.5	*β-actin*	TCCTCTCCCAAGTCCACACAGG GGGCACGAAGGCTCATCATTC	131

control is seen with red diagrams

## Discussion

Stem cells can be of interest in the treatment of different diseases including genetic disease, improving hematopoiesis in cancer patients under treatment, bone regeneration, repairing necrotic tissue in myocardial infarction patients, etc., thanks to their self-renewability properties and their ability to differentiate into various tissues (5, 11). Meanwhile, BM contains HSCs, MSCs, and stromal cells. HSCs residing in the BM communicate with the BM microenvironment and can produce different types of blood cells. MSCs contribute to stimulating the proliferation and differentiation of HSCs to various bloodlines through secreting growth factors, cytokines, and various chemokines such as IL-6, FLT3L, SCF, GCSF, GMCSF, *etc* (23). Thus, direct cell-to-cell contact as well as cytokines secreted from MSCs are involved in the co-culture of mesenchymal cells and HSCs in the hematopoiesis process and determine the fate of HSCs.

Infections are still a major challenge among cancer patients with severe long-term neutropenia following chemotherapy or transplantation with HSCs. With the emergence of granulocyte colony-stimulating factor (GCSF) to mobilize neutrophils, granulocyte injection is widely done to prevent or treat life-threatening infections among patients with neutropenia with high fever or impaired neutrophils (28). 

Meanwhile, injection of granulocytes is associated with some limitations including pyrogenic reactions in response to HLA incompatibility; in case this proposal succeeds, through direct injection of cytokines secreted from MSCs, in stimulating HSCs, which are a part of BM single-nucleus cells in patients with neutropenia (for example, the neutropenic phase before BM transplantation is associated with neutropenia), a significant role can be done towards granulocyte line (28). This is because we know that reduction of granulocytes as the main part of the innate immune system in individuals undergoing chemotherapy or treatments such as BM transplantation is one of the major risks and complications, causing the incidence of numerous problems such as opportunistic infections. Reduction of the duration of renewal of new cells is one of the challenges ahead of medical research, and the positive results obtained from research in this regard can significantly help in driving therapeutic goals and providing newer methods with a more intensive and effective treatment model against malignant tissues.

Meanwhile, other studies have been reported regarding the positive effect of injecting MSCs alongside or together with BM mononuclear cells in cell transplantation. Possibly, the reason behind this positive effect is cytokines and factors secreted from MSCs. This highlights the necessity of the usage of research on cytokines affected or secreted by MSCs (19). The role of MSCs in differentiation to erythroid and myeloid lines is possibly due to the secretion of specific cytokines (29). However, so far the role of cytokines secreted from MSCs in the induction of granulocyte differentiation of HSCs has not been studied. 

Various studies have been performed on the role of MSCs in the differentiation of HSCs. Tocci and Forte (2003) reported that BM-MSCs can maintain long hematopoiesis under *in vitro* conditions (30). Further, the signaling pathways involved in the induction of differentiation of hematopoiesis of HSCs have been examined in several studies. In this regard, pathways such as ERK, Wnt, Notch, SMAD, etc., have been reported as important pathways in self-renewal, differentiation, proliferation, apoptosis, and aging of HSCs. The effective factors in the interaction between MSCs and HSCs have also been reported in other studies. Kikuchi *et al.* (2011) reported that when HSCs are subject to MSCs, the expression of Notch ligands in MSCs increases through the Wnt pathway in HSCs (31). In another study by Simmons and Torok-Storb in 1991, it was found that MSCs in the BM create signals for differentiation and proliferation of HSCs (32). Also, in the study by Moreau *et al.* (2005), the role of cytokines as well as factors secreted and affected by MSCs on HSCs have been reported (33). In the study by Dilva *et al.* in 2005, CD34^+^ HSCs were cultured in a medium fortified with various cytokines in the presence and absence of MSCs (34). It was observed that MSCs supported the growth and differentiation induction of HSCs. In another study by Mehrasa *et al.* (2014), HSCs of the umbilical cord were subjected to co-culture conditions alongside MSCs (35). They found that the MSCs led to high proliferation and reduced apoptosis of umbilical cord HSCs due to secretion of cytokines and various growth factors (35). Meanwhile, another study reported that differentiation of HSCs during their development increases the aging process and cellular death, but this direct contact between cells and MSCs indicates their role in hematopoiesis versus differentiation. More comprehensively based on studies, MSCs produce diverse cytokines and factors affecting the differentiation of HSCs.

In the present study, the results showed that MSCs cause a significant increase in the protein expression levels of JAK3/STAT3, ERK, and PI3K. In a similar investigation by Fathi *et al.* (2021), it was reported that co-culturing of MNCs with BM-MSCs causes an increase in the expression levels of granulocyte markers of MNCs, e.g., CD34, CD16, CD11b, and CD18. Based on these data, one can conclude that MSCs may affect the granulocyte differentiation of MNCs via ERK protein expression (20). 

## Conclusion

Due to problems in the granulocyte injection pathway using GCSF, further research is required regarding alternative pathways. Since MSCs have a supportive role in BM and produce effective growth factors in hematopoiesis, thus there is the possibility of inducing the differentiative effect of these cells on HSCs. In addition, the interaction between two cell lines and the signaling pathways activated resulting from this interaction provides the possibility of proliferation and development of HSCs in the undifferentiated state.

## Authors’ Contributions

EF as the executive of the project had main contribution in conception and design, data analysis, and manuscript writing; IV as the main colleague of the project in providing the kits and some materials needed for this project as well as interpretation of experimental procedure; RF and SAM-N as colleagues involved in performance of experiments, data analysis and manuscript writing. 

## Funding Statment

This work has been supported by the Center for International Scientific Studies & Collaboration (CISSC), Ministry of Science Research and Technology.

## Data Availability Statement

The data sets used and/or analyzed during the current study are available from the corresponding author upon reasonable request.

## Ethics Approval and Consent to Participate

Ethical consent was given by the ethics committee at Tabriz University of Medical Sciences, (Ethics code no: IR.TBZMED.VCR.REC.1397.322) in accordance with the guidelines of the Helsinki-Ethical Principles.

## Conflicts of Interest

The authors declare that they have no conflicts of interest.
